# Engagement of Users in Digital Health Applications: Scoping Review

**DOI:** 10.2196/66002

**Published:** 2026-05-15

**Authors:** Melek Aktas, Linda Cambon, Olivier Aromatario

**Affiliations:** 1 Mérisp Team Labelled League Against Cancer (CIC 1401), BPH (U1219), INSERM, University of Bordeaux, 146 Rue Léo Saignat, Bordeaux, 33000, France; 2 Prevention Unit, University Hospital of Bordeaux, Bordeaux, France

**Keywords:** mHealth, user engagement, participation, digital health interventions, prevention, health promotion, scoping review, health app, behavior change, mobile health

## Abstract

**Background:**

Mobile health (mHealth) uses mobile technology as a tool for prevention and health promotion. Research indicates that user engagement is crucial for effective mHealth interventions and improved health outcomes. However, many studies report low adoption rates, rapid decline after initial use, and a lack of acknowledgment of user implications in achieving outcomes. Thus, conceptualizing participation in mHealth is essential to identify key determinants for engaging users.

**Objective:**

This scoping review aims to identify and characterize the attributes and definitions of user engagement in mHealth, examine engagement methods, and analyze barriers and facilitators influencing participation.

**Methods:**

Following the PRISMA-ScR (Preferred Reporting Items for Systematic Reviews and Meta-Analyses extension for Scoping Reviews) guidelines, Scopus, Web of Science, and PubMed databases were searched for publications between 2000 and 2025 with a 2-stage selection process.

**Results:**

Out of 2489 articles identified, 1416 were screened, and 52 met the inclusion criteria. Half were recently published in the last 5 years (2020‐2025). Existing literature focused on digital interventions for specific populations and health topics. Analysis revealed four main perspectives on engagement in mHealth: (1) usage metrics, (2) subjective user experiences, (3) a hybrid approach that combines both, and (4) a goal-oriented perspective (behavior change or health outcomes).

**Conclusions:**

To understand the complexity and multifactorial nature of participation, it is relevant to conceptualize it as a dynamic mechanism enabling users to achieve their objectives. Both quantitative use and subjective user experience should be integrated to reach the optimal intervention dose. Recognizing users’ evolving needs, uniqueness, and their socioenvironmental context interdependence, it is essential to involve users in all stages (design, implementation, and iterative evaluation of mHealth). Findings will inform an e-Delphi study to establish consensus on engagement criteria.

## Introduction

The World Health Organization’s Global Observatory for eHealth defines mobile health (mHealth) as “medical and public health practices supported by mobile devices such as mobile phones, patient monitoring devices, personal digital assistants (PDAs), and other wireless devices” [1]. mHealth emerged as a promising approach to enhance health care, health promotion, and prevention delivery by leveraging mobile technologies such as smartphones, tablets, and wearable devices [2-5].

Community participation is a crucial element in health prevention and promotion, as highlighted by several important documents and reports. The Ottawa Charter for Health Promotion established community participation as one of the fundamental principles for health promotion [6], and the importance of community engagement in reducing health inequalities has been demonstrated [7]. Going forward, the World Health Organization’s Global Strategy for Health for All by 2030 has also emphasized the importance of community participation in the design, implementation, and evaluation of public health programs [8]. These documents and reports have contributed to establishing the importance of community participation in health prevention and promotion, and encouraged governments and health organizations to partner with communities to design and implement effective health programs. Studies have also demonstrated a correlation between user engagement and the effectiveness of web-based behavior change interventions [9,10]. Evidence suggests that greater user engagement is associated with better health outcomes in various mHealth interventions [11,12].

However, actual usage and sustained engagement with mHealth remain limited. According to data collected between 2010 and 2019 from 12,000 respondents, approximately 25% of downloaded apps were opened only once before being abandoned [13]. More recent findings based on a study of 2200 Android apps revealed that, on average, one out of every two apps is uninstalled within 30 days of initial download, with most of these occurring in the initial 24-hour period postinstallation, often due to unmet expectations or a disconnect between the app’s marketing and the actual user expectation [14,15]. In addition to studying accessibility, it is therefore important to explore the actual usage of these tools [16].

Currently, there is no widely accepted definition of participation [17,18], and there is a variability [19,20] and a lack of clarity around the question of how to conceptualize engagement [21]. Sometimes described as a “suitcase word,” the term engagement can be associated with “almost any action involving the population, to a greater or lesser extent.” Participation can take on various forms and intensities, leading to different classifications in the literature. Comprehensive definitions of participation, its conceptual boundaries, and practical modalities are not always explicit [22]. To our knowledge, there is no consolidated consensus of the characteristics, methods, barriers, and facilitators related to user participation in this context.

Consequently, there is a need to better understand how this concept is mobilized in order to guide future evaluators and designers of digital interventions toward participatory, effective, and sustainable approaches [14].

For these reasons, given the wide and exploratory nature of the research questions and the diversity of the existing literature, a scoping review was conducted in order to identify existing literature on user participation in mHealth. The following research questions were formulated: How is user participation characterized in digital health interventions? What techniques and/or methods are implemented to involve users? What are the barriers and facilitators encountered in implementing user participation?

This paper aims to (1) identify the attributes and definitions of user engagement, (2) examine the methods used for engaging users in mHealth, and (3) identify barriers and facilitators encountered in implementing user participation.

## Methods

### Study Design

A scoping review was chosen as a systematic approach to map the evidence and identify key concepts and relevant theories on the subject. To guide this review, the PRISMA-ScR (Preferred Reporting Items for Systematic Reviews and Meta-Analyses extension for Scoping Reviews) checklist for scoping reviews and tip sheets documents were used [23] (see Checklist 1).

### Eligibility Criteria

Articles eligible for inclusion focused on mHealth interventions targeting health promotion and prevention. All reviews and primary articles were included. The articles had to be published in English or French between January 1, 2000, and April 14, 2025.

Exclusion criteria were studies focused on clinical interventions, disease management, and tool evaluations. Studies on health worker training were also excluded. Based on existing participation research in various other subject areas, interventions that consider engagement as simple service usage (such as logging in), information reception (where users only receive information), and consultation (where users express themselves using free text and this expression has no influence on the process) were excluded [24,25].

### Information Sources

Literature searches were conducted in 3 databases: Scopus, Web of Science, and PubMed. These databases were chosen because of their focus on social behavior and medical sciences. Keywords were collected from articles of interest until saturation was reached. Then, keywords were refined considering the vocabulary of each database, adding additional keywords, using truncation to search by title, and sometimes by title and abstract. The search strategy was different for each database, adapted to the respective databases, and included the following terms divided into 3 categories relating to subject, medium, and field (see Multimedia Appendix 1).

### Selection of Sources of Evidence

Search results were uploaded to Zotero to sort out duplicates. Then, the articles were transferred to Covidence, a web application for independent selection of articles by multiple researchers. A 2-stage selection process was established. First, titles and abstracts were screened by two authors (MA and OA) for inclusion or exclusion of articles. Then, discrepancies were solved through discussion until a consensus was reached. Finally, a full-text screening was carried out (MA).

### Data Items and Synthesis of Results

The selected articles were analyzed using an evolving reading grid in Microsoft Excel, which includes a list of themes of interest based on the aim of the scoping review and research questions. These themes included (1) article presentation (goals, target population, etc); (2) attributes of participation (definitions, scales, etc); (3) methods used to engage users (theory, model, framework, etc); and (4) barriers, facilitators, and strategies to promote engagement.

## Results

### Overview

**Figure 1.**
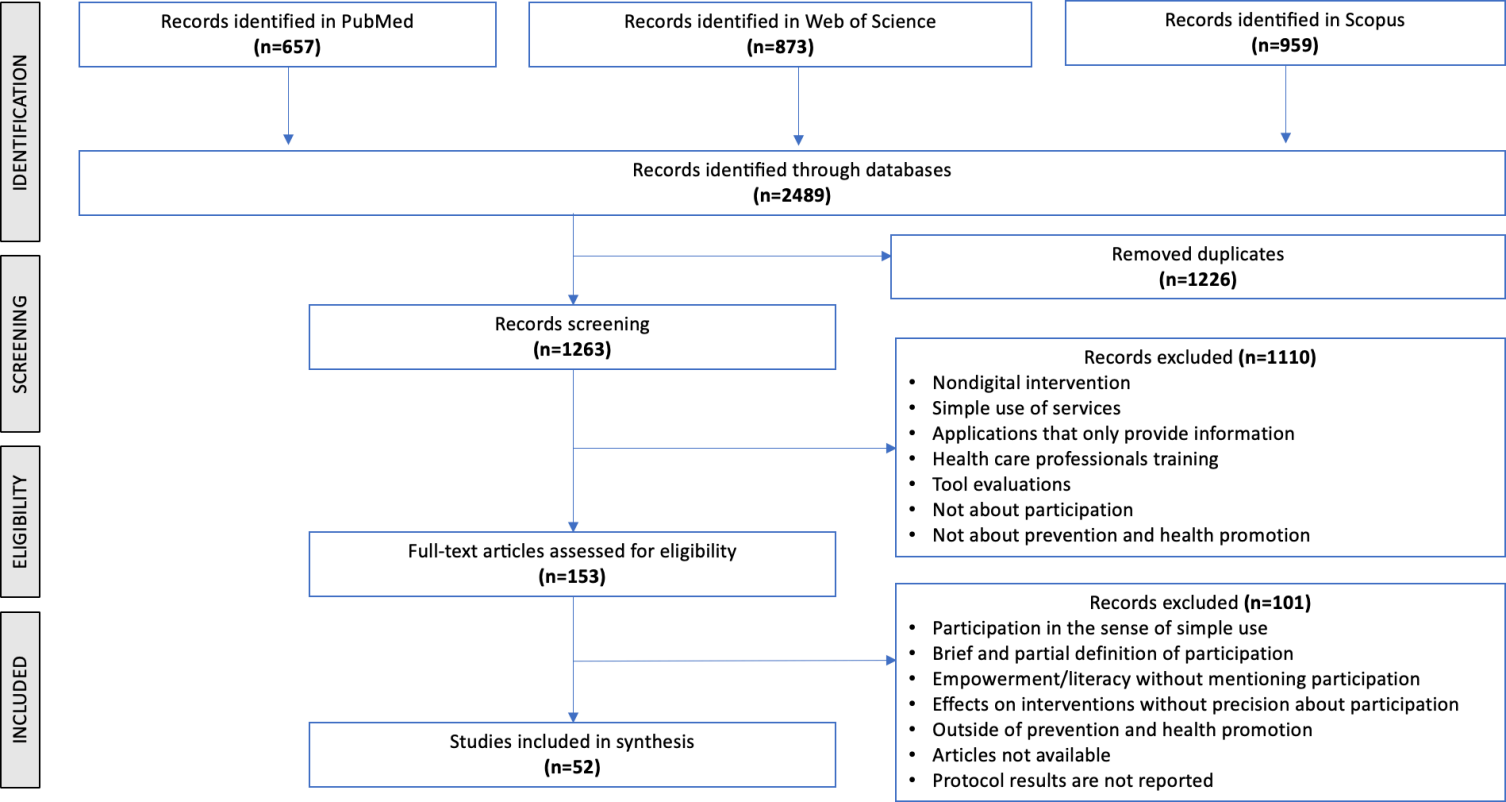
Flowchart of the article selection process.

A total of 2489 articles were identified, 1226 duplicates removed, resulting in 782 unique articles. After reviewing the titles and abstracts, 153 articles were selected to read in full. Following a review of the complete articles and application of exclusion criteria, 52 articles were finally selected for analysis. Our selection process is illustrated in a flowchart (Figure 1).

### Description

A total of 52 articles were analyzed in this study. The chronological distribution indicates that 25 (48%) articles were published in the last 5 years (2020‐2025), which corresponds to nearly the same number as those published over the previous 20 years (2000‐2020), with 27 (52%) articles. Of the articles reviewed, 39 focused on digital interventions for a specific target audience, with 14 of these specifically aimed at adolescents and young adults. The 16 articles that are not about a digital intervention for a target audience are thematic articles that do not address a specific digital health intervention. In terms of health topics, 40 articles focused on a specific health issue, with prevention and/or health promotion being the most common topic in 7 articles, followed by AIDS in 5 articles, obesity in 4 articles, and tobacco in 3 articles (see Multimedia Appendix 2) [10,14,17-21,26-70].

Depending on the article, some or all of the information regarding definitions and attributes of participation, scales and methods of assessment, techniques used to involve users, and facilitators and barriers to implementing participation were obtained (see Multimedia Appendix 3) [10,14,17-21,26-70].

The results are organized into the following main subcategories to guide interpretation: (1) engagement attributes and definitions, (2) scale and gradation of participation, (3) methods and techniques used to engage users in digital health interventions, (4) difficulties and barriers to engagement, and (5) facilitators and suggestions to support sustained participation.

### Engagement Attributes

First, there are different attributes depending on the discipline: according to scientists, participation is adherence and satisfaction, while for computer scientists, it corresponds to increased attention and enjoyment [21]; in behavioral literature, participation is frequency or duration, while for the gamification industry, it is subjective experience with the service including affect, interest, attention, and flow [18,26].

One recurring way of conceptualizing engagement is the duration and frequency with which the participant uses the eHealth intervention [10,21,27]. In the literature, engagement is generally considered to be ease of use, measuring interactions with the features and functions of the digital solution [17,18]. Engagement with digital health interventions can be defined by using quantity (eg, total number of times the intervention was accessed), duration (eg, total duration of use), frequency (eg, patterns of use), and depth (eg, content consumed during the intervention) [28,29].

However, when it comes to the frequency or duration of time spent using a digital service, some studies suggest that low frequency of use can be just as effective as higher frequency [26], and an increase in usage time can signal low ease of use rather than attractive content [20]. In the context of mHealth, engagement behavior is frequently assessed through passive measures of application usage [30], which fail to provide a comprehensive understanding of engagement with a digital platform [19]. Additionally, there is ongoing discourse surrounding the definition of this “more than usage” [18]. A study indicates that it is important to focus on “effective engagement” rather than simply “increased engagement” [14]. In 3 articles, the authors differentiate between engagement (meaning interactions with the tool) and effective engagement (sufficient participation in the intervention to achieve the desired effects) [14,20,31]. To achieve these desired effects, there is a need to determine an optimal duration and frequency of engagement [20]. However, the concept of optimal engagement dose is not yet clear in the field of digital health interventions [14,18]. In addition, the concept of participation is often confused with compliance, which refers to an individual following an instruction and using the intervention as intended by the developers [20,21,32].

In addition, to characterize participation, some studies focus on the user’s experience, but they do not define experience in the same way [21]. For some, user experience is about interaction [17,19] or the desire of a user to use the application for longer and at frequent intervals. User experience quality is characterized by attributes of challenge, positive affect, endurance, aesthetic and sensory appeal, attention, feedback, variety/novelty, interactivity, and perceived user control [33] and includes sensual (aesthetic or novel elements that promote attention and interest), emotional (positive or negative effect elicited), and spatiotemporal (perceptions and awareness of time and the physical environment that the user experiences during use) categories [10].

Although authors assert that the subjective user experience can provide information about the degree of immersion in an application, it does not generally explain the cognitive engagement in the behavior change process. A health application can be entertaining and have good usability, but it does not necessarily lead the user to reflect on their behavior or to take action toward achieving their goals [26].

In one study, to define participation, the aforementioned elements of usage and experience are combined. Engagement in digital interventions is defined through the measures of usage extent (eg, quantity, frequency, duration, and depth) and subjective experience (eg, attention, interest, and affect) [14,34,35]. In the field of digital health behavior change interventions, engagement should therefore integrate objective measures (defined based on usage habits measured using various tools to track the number of logins, time spent online, and amount of content used during the intervention period, and also physiological measures using wearable sensors such as heart rate and electrodermal activity, as well as eye-tracking to determine psychophysiological measures) and subjective measures (defined based on self-evaluation questionnaires measuring levels of engagement with digital games and the intervention) [14,20].

Finally, the literature includes some definitions focused on the objective to be achieved: health benefit [32,36], changes for benefit [37], or behavior change [38]. One source defines participation in eHealth and health behavior change as “the process of involving users in health content in a way that motivates and drives health behavior change,” while specifying that this is influenced by a number of variables [14,37-39] and is a complex process because it is evolving [39]. Engagement itself is influenced by intervention characteristics such as content, delivery mode, and contextual characteristics such as physical environment and individual characteristics; there are also potential moderators and mediators of the engagement process [21]. This multidimensional aspect is also mentioned in several articles, where engagement is described as a multidimensional process that involves, among other things, cognitive, emotional, behavioral, and social dimensions [27,30,33,36,40]. One study examined 3 fundamental dimensions: behavior, cognition, and affect. At the behavioral level, the successful usage of a given system is characterized by its routine integration into the daily lives of its users, ease of use, and intuitive interface design that requires minimal effort, and dynamic adaptability to meet the needs of users in different contexts or periods. From a cognitive perspective, mHealth must function as an effective instrument to facilitate the achievement of objectives, while concurrently offering novel perspectives through the provision of pertinent, personalized information, thereby sustaining motivation over an extended period. Finally, on an affective level, a positive experience translates into the pleasure of making progress, general satisfaction in using the application, and a feeling of attachment to the intervention, to the point of feeling its absence when it is not available [18]. Thus, for evaluation, engagement is too complex to be evaluated by a single method, and a combination of several methods should be used [21,41]. The advent of novel technologies has precipitated the application of artificial intelligence, and more specifically, machine learning techniques, in the measurement of a diverse array of engagement. These phenomena encompass goal setting, learning, problem-solving, affect, and reflection, among others [40].

### Scale and Gradation of Participation

The gradation of participation in articles is present in two different ways: either within the various stages of the process or through the establishment of a gradient that characterizes a level of engagement, ranging from low to high.

The conceptual model in Yardley et al [71] distinguishes between the micro and macro levels of engagement. *Micro-engagement* refers to the user’s immediate interactions with the technology’s features, including the extent of intervention use (eg, number of completed activities) and the user’s experience (eg, level of interest and attention during activity completion, specifically linked to attention, interest, and affect). *Macro-engagement*, on the other hand, refers to how the user engages with the overall goal of behavior change. After a period of effective micro-engagement, the user may disengage from the platform while still being immersed in the behavior change process [10,18,21,28,34]. Another similar method is proposed by Cole-Lewis and colleagues, defining *Big E* as engagement in the targeted health behavior, and *little e* as engagement in the digital intervention for behavior change [17,40,42].

Regarding scales of participation ranging from least engaged to most engaged, scales are identified in some articles, but they are not specific to digital health interventions [43-48]. First, the most common participation scale is Arnstein’s 8-rung ladder of citizen participation, ranging from nonparticipation in citizen power through the stages of manipulation, information, consultation, advocacy, partnership, delegation, control, and self-management [43]. Additionally, there are classifications in terms of usage quantity: an active user may post on a forum, for example, whereas a passive user may read an informative article [44]. Alternatively, a heavy user is one who uses the intervention several times a day or 1‐2 days per week, and a light user is one who uses the intervention twice per month or once per month, or who has not used it in the past month [45]. There is also a 4-position psychological scale on a continuum ranging from minimum to maximum engagement [46], patient participation levels at 3 levels in the health care sector [32], a 4-stage scale representing the developmental nature of patient activation [47], and other specifically designed questionnaires for a study [47,48]. Taki et al, on the other hand, suggest a mathematical indicator of engagement that uses 5 different indices to assess user engagement based on usage [20,72].

User typologies are also found. One study classifies users based on their alcohol consumption: “trackers” monitor and track their alcohol consumption, “cut-downers” intend to reduce their alcohol consumption (similar to trackers, they used not only the monitoring features of the application but also goal-setting and feedback functions), and “noncommitters” lack the motivation to use health applications [10]. Another study categorizes users into 3 groups based on their frequency of engagement in peer interactions: “conversation starters,” who initiated the highest number of threads; “frequent posters,” who posted the highest number of messages on forums; and “conversation attractors,” whose messages received the most responses [19].

### Methods/Techniques Used to Engage Users

Many authors emphasize the need for theory-based interventions in order to engage users [34]. A theory-based intervention is a systematic approach that uses theoretical models to understand how behaviors are influenced by factors such as beliefs, attitudes, and social norms. By applying these theories to the design of digital health interventions, designers can create programs that specifically target these factors, ensuring that they are based on solid data and effectively encourage user participation [28,46,49]. These theories can be specific to health behavior change models or specific to digital interventions [28]: derivatives of behavioral and neurocognitive theories and models such as the theory of planned behavior, social cognitive theory, self-regulation theory, and social learning theory or unified theory of acceptance and use of technology, which is a theoretical model to explain the adoption and use of technology by users [49,50].

Similarly, the use of appropriate behavior change techniques (BCTs) can also increase user engagement and help them maintain healthy behaviors in the long term. This can improve the overall user experience and make digital health interventions more effective. BCTs are the smallest active components of an intervention that are capable of changing behavior [31,34,51]. The 6 most common BCTs associated with engagement were social support, goal setting, feedback, prompts/cues, self-monitoring, and rewards [31,34]. However, the author claims that they depend on their design, dose, and duration, as well as their content, to optimize their effectiveness. The most advanced and complete BCT would be useless if it did not generate interest through an attractive design, or if it was not user-friendly and intuitive to use in terms of digital functionalities [33].

Behavioral incentive strategies, including action planning, prompts, and habit formation, have been demonstrated to promote sustained use. It is imperative that techniques such as goal setting with visualization and tracking of progress [52], self-monitoring, biofeedback, and behavioral feedback are prioritized for development. Furthermore, providing a range of rewards and incentives that align with distinct core values has been demonstrated to foster sustained engagement, given the recognition that user responses to incentives vary [51]. In addition, one of the most widespread methods for engaging users is user involvement in the intervention design, a process that takes different names such as user-centered design [37,42,53,54], cocreation [55-58], codevelopment [52], or participatory design [46,59]. It is important to involve end users from the beginning of the process to ensure that the intervention is relevant and accepted by users in order to trigger effective participation afterwards [45,60,61] and increase technology literacy and access [52]. Users were also invited to implement and/or evaluate the intervention [42,62,63]. Some authors mentioned that the process is spiral and works in a loop: ideas are generated by/with the community, implemented by the community, evaluated by the community, adjusted based on community feedback, and then implemented and evaluated [41,42,54,64].

Apart from being used for co-design, personalization is a technique used on its own to engage users [60,65]. Some articles mention the need to gather individuals’ needs to tailor and personalize interventions in order to engage end users [14,27,30,46,60,66], for example, in adapting content by personalizing it according to variations in psychological, social, and behavioral profiles [28,50] or based on user typologies determined during application registration (eg, through a short questionnaire and targeting the application’s content according to their typology) [10]. It is imperative to ensure that the data accurately reflects the individual and their situation in order to guarantee the credibility and accuracy of information and feedback [51,58]. The overall goal is to provide instant contextual support for targeted behaviors when the individual is most likely to be receptive. Just-in-time adaptive interventions could use sensory data, for example, a smartphone or smartwatch, and momentary information directly from participants [60].

Thus, autonomy is also one of the techniques used to engage users [30]. In a structured and self-directed behavior change intervention, all participants receive the same content, but each individual is encouraged to choose their own behavioral goals and activities [47,67]. In order to provide specific recommendations based on individuals’ needs, personalized feedback can be used [27,28,48].

Regarding users, some authors also suggest using online peer communities to engage users as they provide resources for sharing experiences and achieving better outcomes. These digital health communities may include discussion forums, online support groups, and social networks for patients [27,30,32,41,43,50,65,68,69]. These digital health communities are particularly important for young people and marginalized populations as they provide an opportunity to address crucial behavior change issues [69] and allow social interaction, feeling supported, and understood by the community [19,28,66] to enhance participants’ engagement in the program and foster their motivation [27], mentioned as a key to engaging users [26,30,33,39]. Regarding this, despite evidence of improved intervention engagement in physical interventions, motivational support such as motivational interviewing is rarely present in health apps for smartphones. One author indicates that the reason for the limited use of motivational interviewing may be its lack of a coherent theoretical framework and proposes that self-determination theory can be used as a theoretical basis because it shares fundamental principles with motivational interviewing [26]. Self-determination theory, used by several authors, emphasizes autonomy, competence, and social relatedness to encourage user participation [26,41,65].

While motivational interviewing provides tools for self-reflection and satisfaction, gamification can provide experiences of autonomy, competence, and relatedness by adding fun and excitement to activities [14,26,28,43,55,59], and gamification can enhance hedonic and intrinsic motivation by addressing the psychological and emotional requirements of individuals through the use of game-like components [45,58]. Intrinsic motivation refers to performing an activity solely for pleasure, excitement, and interest. Authors assert that gamification of health applications is a promising approach to counter the often-decreasing long-term motivation of health application users [45].

Finally, emotion has been identified as a key mechanism of action that can engender user experience linked to engagement. Ensuring high user satisfaction with mHealth and “avoiding negative emotions” such as frustration, disappointment, or anxiety should result in good long-term engagement and an increase in the number of positive reviews [31,51].

### Difficulties in Engaging Users

Authors encountered several barriers to user engagement in digital interventions. At the country level, in several countries and populations, technological adoption has been slow due to cost, infrastructure, design models, architecture, integration, usability, and the implementation of public policies. Adoption and deployment are also dependent on the training and education of users provided by authorities [32,51].

At the intervention level, difficulty in involving all populations in participatory design is encountered due to knowledge asymmetry [63]. Lack of user involvement in the design and a lack of desired features lead to reluctance to adopt the intervention [45,65,66]. In the long term, maintaining usage is also a recurring problem [14,45,64]: there is evidence indicating that people often download an app and never use it again, with a rapid decline in usage after the first download [73,74]. Research on the specific reasons for user abandonment and low engagement in online programs has identified a lack of motivation, an absence of goal tracking, and negative feedback when goals are not met [52].

Regarding the content of interventions, there is a lack of theory-based mHealth interventions, despite the fact that some behavior change theories have been well-validated and tested in evidence-based prevention, diagnostic, and care interventions [61]. As for interfaces, some studies emphasize that interfaces are non–user-friendly [29,43,50] and that it is challenging to compete with social media and other entertainment-based applications [28,60]. As for all other online tools, data security is also a recurring concern, particularly with respect to anonymity [43,50]. Once the results of an intervention are obtained, the literature mentions the difficulty of generalizing the results to other populations due to differences in context. What works in one context for a given subgroup of the population may be less effective or even harmful elsewhere for other subgroups [37].

At the user level, technical barriers and a lack of technological skills are cited as a hindrance to participation [42,43,66]. In addition, improving health literacy directly impacts the ability to act on health information and take greater control of health as individuals, families, and communities, and is considered an essential condition for patient participation [32,36,50,51]. Engagement also varies according to sociodemographic characteristics; young women and individuals with a higher level of education are more involved [50]. Additionally, lack of support [43,66] and lack of motivation were associated with low adoption of mHealth apps [45].

### Facilitators and Suggestions to Engage Users

At the user level, engagement depends on several interdependent components such as social norms, positive and negative reinforcement factors, goal-setting, self-monitoring, self-evaluation [17,49], self-efficacy [70], social support [19,49,67], motivation [34,45], trust and adherence of the participants’ family, and peer modeling [37,50], especially through social media and friends or any scientific authority [45,58]. In addition, the delivery of human support has been demonstrated to positively influence engagement with digital behavior change interventions and increase adherence with mHealth interventions. Support personnel like health coaches provided individualized and personalized support, and facilitated comprehension and application of BCTs, including goal setting and receiving feedback on behaviors that had been monitored [31].

The social facilitators associated with the intervention encompass the following factors: the establishment of positive relationships and effective communication, the development of trust in the intervention’s leader, the usage of the program within a social context, and the cultivation of positive connections with fellow participants. The temporal and spatial flexibility facilitators of the intervention are characterized by their accessibility and adaptability to different environments (performed within the comfort and familiarity of the participant’s home, thereby eliminating the need for travel) [70].

Regarding intervention features, gamification [19,26,33,45,51,59], accessibility and ease of use [14], interactivity [67], attractiveness [55,60], confidentiality and security of personal data [14], and adapting the material for potential users from socially or educationally disadvantaged backgrounds [37] all help users to engage and are avenues to explore to increase user engagement within digital health interventions. For instance, the configuration of push notifications could be adapted to align with the preferences of the end user, or alternatively, users have the option to disable them entirely. The hypothesis was that these customizations would provide a more pleasant user experience and facilitate the daily use of the mHealth apps [50,58].

Regarding the design of the interventions, results show that involving users at all stages [26,41], addressing basic psychological needs [26] including psychosocial determinants anchored in relevant behavioral theories, such as the social ecological model, the COM-B (capability, opportunity, motivation—behavior) model, and social cognitive theory [17], using more systematic and progressive approaches for intervention development and evaluation [48], integrated skill acquisition, positive reinforcement, integration of cultural values and group norms [49] could be explored. One study suggests letting people address their own problems in their own way, but within a structured and supportive framework [67].

A conceptual synthesis of the determinants influencing user engagement in digital health interventions, as identified in this review, is presented in Multimedia Appendix 4.

## Discussion

### Principal Results

This paper identified the attributes and definitions of user engagement, examined the methods used for engaging users in mHealth, and identified barriers and facilitators encountered in implementing user participation. In the literature, a variety of information was found in the articles. Some articles contained information in all the categories (definition or attributes, scale or gradation of participation, methods, and facilitators or barriers of participation), while others provided detailed information only on one category. In 18 articles, the authors mention attributes or a definition concerning participation. Engagement in digital health interventions is described as multidimensional, encompassing both objective measures (eg, usage frequency, duration, and depth) and subjective experiences (eg, attention, interest, and affect), as well as the goal of behavior change. Methods used to involve users are the most frequently cited, with 38 articles. Main methods and techniques used are theory-based intervention design, BCTs, personalization, user-centered and participatory design, peer support, autonomy, gamification, and emotion management. The obstacles encountered in involving users are discussed in 19 articles. Difficulties in user engagement in digital health interventions arise from technical, contextual, and individual barriers, while facilitators include participatory approaches, personalization, gamification, social support, and human guidance to enhance motivation, adherence, and sustained use. Some articles deal with digital interventions in general, while others are based on one specific digital intervention. The literature on this topic is recent, with half of the articles selected dating from the last 5 years.

### Combining Approaches to Define Participation

The characterization of participation in digital health interventions for health promotion and prevention is complex [39] and influenced by different factors [40]. This is consistent with the complexity of digital health interventions themselves, which often involve multiple components [14,21,27,30,37-39]. Addressing this complexity requires integration of multiple approaches. The first approach is a quantitative perspective, which conceptualizes participation through measurable indicators such as duration and frequency, focusing on the extent of tool usage [10,17,21,27,28]. The second is a user experience approach, which captures subjective dimensions including attention, interest, emotions, and perceptions [19,24,28,35]. Combining these approaches enables a more comprehensive understanding of effective user engagement with mHealth tools in achieving behavior change goals of users [32,36-38]. In this context, user participation in the intervention becomes a prerequisite for the involvement of the user with their behavior change objective [17,71]. This engagement is possible when users consume an optimal intervention dose [20]. Engagement can thus be conceptualized as a continuum, ranging from minimal involvement to an excessive, potentially counterproductive level. The aim is to identify and deliver the appropriate dose of engagement for each individual, thereby enabling the achievement of their behavior change goals.

### Participation Is the Goal but How Can It Be Reached?

To achieve engagement, the literature mentions various methods and techniques in three levels: (1) user-related factors, (2) intrinsic characteristics of the intervention, and (3) implementation context. It is therefore essential to have a holistic vision to ensure the effective condition of participation.

#### User-Related Factors

Thus, mHealth interventions should enable individuals to enter into a process that allows them to make choices aimed at maintaining or improving their health status, as stated in the Ottawa Charter [6]. This means (1) being able to improve their individual ability to act through the use of effective BCTs such as Michie’s [75,76] BCTs, (2) engaging in collective discussions that correspond to the needs and demands of users, (3) providing social support such as the collective discussion that founded in this review, (4) becoming aware of the effect of their environment on their behavior and being able to act on it [27,30,32,41,43,65,68,69], and (5) finding external human resources that improve the effectiveness of applications [77].

#### Intrinsic Characteristic of Intervention

To better understand users’ needs and involve them in the development process, one of the most commonly used methods for engaging users is cocreation, which involves users in the creation phase of the tool. This cocreation is even more valuable because one of the reasons for the long-term decline in tool usage is the absence of functionalities that users need. However, it is not sufficient to simply include users in the development process, as the mHealth functionalities must be designed to evolve with users’ needs and demands. The process is a spiral and works in a loop [41,42,54,64]. Users’ needs vary significantly over time, and these changes are influenced by many factors, such as life events, health experiences, and interactions with the health care system. Therefore, it is advisable to monitor users’ evolving needs over time to adapt the digital intervention accordingly and meet the changing needs of users [18,78]. So, engaging users in just one step is insufficient. Perhaps, it is necessary to rethink the design methods of applications that currently attempt to suit everyone, and which, as a result, are suited to no one, even if they are intended for a targeted population group. The design should be based on a user-centered approach that incorporates cocreation methods and iterative testing. It should also offer possibilities for adaptive personalization based on behavioral profiles.

Engagement techniques are grounded in robust theoretical models, such as the COM-B framework, and can be bolstered by gamification strategies and interactive features that provide personalized feedback. The overall digital experience should combine an intuitive and ergonomic interface for ease of use with careful design for attractiveness.

#### Implementation Context

Data security is also a recurring concern in terms of the functionality of the intervention [43]. Furthermore, once the results of the intervention have been obtained, there may be difficulties in generalizing the results to other populations due to differences in context: what may work in a given context for a given subgroup of the population may be less effective or even harmful elsewhere for other subgroups [37]. In addition, engagement is frequently subjective, and the factors that motivate one user to repeatedly use an application may not be applicable to another user, or may not be applicable at a specific instance [35].

These 3 different levels interact dynamically to create a complex mechanism. For example, a poor user experience can reduce motivation and lead to the intervention being abandoned prematurely [79,80], whereas a favorable sociocultural context can reinforce long-term engagement [81].

### Conclusions

The current state of knowledge allowed us to have a vision of the participation of users in digital interventions. However, the concept of participation, which is described as complex, evolving, and multifactorial, is a notion on which there is as yet no consensus. This paper offers insights into user participation in mHealth, as evidenced by the results of the review. Usage of the tool, combined with the user’s experience of it, serves as an indicator of participation. This participation is a prerequisite for the user to receive the optimal dose of intervention content, thereby supporting the engagement of users with their behavior change objectives. To foster such engagement, a variety of methods can be mobilized. These methods fall into 3 key categories: user-related factors, intrinsic characteristics of the intervention, and the context of implementation. Such clarifications could then inform the methodology for evaluating and developing digital interventions. In order to address this gap, the results of this review are used in an e-Delphi study with the aim of establishing a consensus definition with experts. The results of this forthcoming study will be the subject of a subsequent publication.

## Supplementary material

10.2196/66002 Multimedia Appendix 1 Search equations and keywords.

10.2196/66002 Multimedia Appendix 2 General characteristics of articles.

10.2196/66002 Multimedia Appendix 3 Information contained in the articles.

10.2196/66002 Multimedia Appendix 4 Conceptual synthesis of the determinants influencing user engagement in digital health interventions.

10.2196/66002 Checklist 1 PRISMA-ScR checklist.

## Abbreviations

BCT: behavior change technique
COM-B: capability, opportunity, motivation—behavior model
mHealth: mobile health
PRISMA-ScR: Preferred Reporting Items for Systematic Reviews and Meta-Analyses extension for Scoping Reviews
