# Supplementary material for: Engagement of Users in Digital Health Applications: Scoping Review
Source: JMIR Mhealth Uhealth. 2026 May 15;14:e66002. doi: 10.2196/66002 (PMC13179053; doi:10.2196/66002)
Supplement: Multimedia Appendix 1 [file mhealth-v14-e66002-s001.pdf]

SEARCH EQUATIONS AND KEYWORDS

The following table categorizes and lists the keywords used to construct the search equations.

SUBJECT	MEDIUM	FIELD
• Participat*<br>• Involv*<br>• İmplicat*<br>• Empower*<br>• Activat*<br>• Engag*<br>• Collaborat*	• Internet Based<br>• Internet-Based<br>• Web based<br>• Web-based<br>• Online<br>• Internet<br>• Mobile App*<br>• Smartphone App*<br>• Mobile Health<br>• mHealth<br>• m-health<br>• m health<br>• Digital*<br>• Mobile phone*<br>• Cell phone*<br>• Social media*<br>• Telehealth<br>• Tele health<br>• eHealth<br>• e-health<br>• e health	• prevention<br>• Health education<br>• Health promotion<br>• Social behaviour<br>• Health behaviour<br>• Consumer behaviour

Search equations used in the 3 databases;

For PubMed:
(((participat*[Title] OR involv*[Title] OR implicat*[Title] OR empower*[Title] OR activat*[Title] OR engag*[Title] OR collaborat*[Title]) AND ("internet based"[Title] OR "internet-based"[Title] OR "web based"[Title] OR "web-based"[Title] OR online[Title] OR internet[Title] OR "mobile app*"[Title] OR "smartphone app*"[Title] OR "mobile health"[Title] OR mhealth[Title] OR m-health[Title] OR "m health"[Title] OR digital[Title] OR "mobile phone"[Title] OR "cell phone"[Title] OR "social media*"[Title] OR telehealth[Title] OR "tele health"[Title] OR ehealth[Title] OR e-health[Title] OR "e health"[Title])) AND (prevention[Title/Abstract] OR "health education"[Title/Abstract] OR "health promotion"[Title/Abstract] OR "social behaviour"[Title/Abstract] OR "health behaviour"[Title/Abstract] OR "consumer behaviour"[Title/Abstract])) AND (("2000/01/01"[Date - Publication] : "3000"[Date - Publication]))

For Scopus:
(TITLE (participat* OR involv* OR implicat* OR empower* OR activat* OR engag* OR collaborat*) AND TITLE ("internet based" OR "internet-based" OR "web based" OR "web-based" OR online OR internet OR "mobile app*" OR "smartphone app*" OR "mobile health" OR mhealth OR m-health OR "m health" OR digital* OR "mobile phone*" OR "cell phone*" OR "social media*" OR telehealth OR "tele health" OR ehealth OR e-health OR "e health") AND TITLE-ABS (prevention OR "health education" OR "health promotion" OR "social behaviour" OR "health behaviour" OR "consumer beahviour")) AND PUBYEAR > 1999

For WEB OF SCİENCE:
TI= (participat* OR involv* OR implicat* OR empower* OR activat* OR engag* OR collaborat*) AND TI= ("internet based" OR "internet-based" OR "web based" OR "web-based" OR online OR internet OR "mobile app*" OR "smartphone app*" OR "mobile health" OR "mhealth" OR "m-health" OR "m health" OR digital OR "mobile phone*" OR "cell phone*" OR "social media*" OR telehealth OR "tele health" OR ehealth OR e-health OR "e health") AND TS= (prevention OR "health education" OR "health promotion" OR "social behaviour" OR "health behaviour" OR "consumer behaviour")
