# Supplementary material for: Engagement of Users in Digital Health Applications: Scoping Review
Source: JMIR Mhealth Uhealth. 2026 May 15;14:e66002. doi: 10.2196/66002 (PMC13179053; doi:10.2196/66002)
Supplement: Multimedia Appendix 2 [file mhealth-v14-e66002-s002.docx]

Information Contains in the Articles

The table below provides a list of the contents of the 52 articles that were analyzed as part of the scoping review.

| First author | Contains a definition or attributes of participation | Contains a scale of participation | Contains evaluation for participation | Contains methods for engaging | Contains facilitators for participation | Contains barriers to participation |  |
| --- | --- | --- | --- | --- | --- | --- | --- |
|  |  |  |  |  |  |  |  |
| Kwann P [49] |  |  |  | ✓ | ✓ |  |  |
| Cole-Lewis H [17] | ✓ | ✓ |  |  | ✓ |  |  |
| DeSmet A [53] |  |  |  | ✓ |  |  |  |
| Silva CC [59] |  |  |  | ✓ | ✓ |  |  |
| Marin HF [32] | ✓ | ✓ |  | ✓ |  | ✓ |  |
| Craig Lefebvre R [38] | ✓ |  |  | ✓ |  |  |  |
| Chen Z [67] |  |  |  | ✓ |  |  |  |
| Kim M [39] | ✓ |  |  | ✓ |  |  |  |
| Corbett T [66] |  |  |  | ✓ |  | ✓ |  |
| Baltierra NB [44] |  | ✓ |  | ✓ |  | ✓ |  |
| Fulton EA [33] | ✓ |  |  | ✓ |  |  |  |
| Alshurafa N [20] | ✓ | ✓ | ✓ |  |  |  |  |
| Hightow-Weidman LB [28] | ✓ | ✓ |  | ✓ |  | ✓ |  |
| Graffigna G [46] |  | ✓ |  | ✓ | ✓ |  |  |
| Levine D [68] |  |  |  | ✓ |  |  |  |
| Ronen K [69] |  |  |  | ✓ |  |  |  |
| Livingood WC [62] |  |  |  | ✓ |  |  |  |
| Van Bruinessen I [65] |  |  |  | ✓ | ✓ | ✓ |  |
| Pernencar C [55] |  |  |  | ✓ |  |  |  |
| Short CE [21] | ✓ |  | ✓ |  |  |  |  |
| Lawrence K [42] |  | ✓ |  | ✓ |  | ✓ |  |
| Wagner B [30] | ✓ |  | ✓ | ✓ |  |  |  |
| Skinner H [41] |  |  |  | ✓ |  |  |  |
| van Hierden Y [56] | ✓ |  |  | ✓ |  |  |  |
| Schroeer C [43] |  | ✓ |  | ✓ |  | ✓ |  |
| Rai T [37] | ✓ |  |  | ✓ | ✓ | ✓ |  |
| Nurmi J [26] | ✓ |  |  | ✓ | ✓ | ✓ |  |
| Mustafa AS [45] |  | ✓ |  | ✓ |  | ✓ |  |
| Morisson L [48] |  |  | ✓ | ✓ |  | ✓ |  |
| Partridge SR [60] |  |  |  | ✓ |  | ✓ |  |
| Mauka W [54] |  |  |  | ✓ |  |  |  |
| Solomon M [47] | ✓ | ✓ | ✓ | ✓ |  | ✓ |  |
| Nitsch M [27] | ✓ |  |  | ✓ |  | ✓ |  |
| Sucala M [64] |  |  |  | ✓ |  | ✓ |  |
| Laidlaw R [57] |  |  |  | ✓ |  |  |  |
| Toefy Y [61] |  |  |  | ✓ |  | ✓ |  |
| Njie-Carr VPS [63] |  |  |  | ✓ |  | ✓ |  |
| Myneni S [19] | ✓ | ✓ | ✓ | ✓ | ✓ |  |  |
| Musso M [36] | ✓ |  |  | ✓ |  |  |  |
| Milward J [10] | ✓ | ✓ |  | ✓ |  | ✓ |  |
| Saleem M [14] | ✓ |  | ✓ | ✓ |  | ✓ |  |
| Hawkes RE [31] | ✓ |  | ✓ |  | ✓ | ✓ |  |
| Kelders SM [18] | ✓ | ✓ | ✓ |  |  |  |  |
| Turcotte S [70] |  |  |  |  | ✓ | ✓ |  |
| Grieve N [29] | ✓ |  |  |  |  | ✓ |  |
| Milne-Ives M [34] | ✓ |  | ✓ | ✓ |  |  |  |
| Mair JL [51] |  |  |  | ✓ | ✓ | ✓ |  |
| White BK [35] | ✓ | ✓ | ✓ |  |  |  |  |
| Giovanelli A [40] | ✓ |  | ✓ | ✓ |  |  |  |
| Isakadze N [52] |  |  |  |  | ✓ | ✓ |  |
| Dederichs M [58] |  |  |  | ✓ | ✓ | ✓ |  |
| Ho TQA [50] | ✓ |  | ✓ | ✓ | ✓ | ✓ |  |
