# Supplementary material for: Engagement of Users in Digital Health Applications: Scoping Review
Source: JMIR Mhealth Uhealth. 2026 May 15;14:e66002. doi: 10.2196/66002 (PMC13179053; doi:10.2196/66002)
Supplement: Multimedia Appendix 3 [file mhealth-v14-e66002-s003.docx]

Information Contains in the Articles

The table below provides a list of the contents of the 52 articles that were analyzed as part of the scoping review.

| First author | Contains a definition or attributes of participation | Contains a scale of participation | Contains evaluation for participation | Contains methods for engaging | Contains facilitators for participation | Contains barriers to participation |  |
| --- | --- | --- | --- | --- | --- | --- | --- |
|  |  |  |  |  |  |  |  |
| Kwann P [50] |  |  |  | ✓ | ✓ |  |  |
| Cole-Lewis H [16] | ✓ | ✓ |  |  | ✓ |  |  |
| DeSmet A [54] |  |  |  | ✓ |  |  |  |
| Da Silva CC [60] |  |  |  | ✓ | ✓ |  |  |
| De Fatima Marin H [33] | ✓ | ✓ |  | ✓ |  | ✓ |  |
| Craig Lefebvre R [39] | ✓ |  |  | ✓ |  |  |  |
| Chen Z [68] |  |  |  | ✓ |  |  |  |
| Kim M [40] | ✓ |  |  | ✓ |  |  |  |
| Corbett T [67] |  |  |  | ✓ |  | ✓ |  |
| Baltierra NB [45] |  | ✓ |  | ✓ |  | ✓ |  |
| Fulton EA [34] | ✓ |  |  | ✓ |  |  |  |
| Alshurafa N [19] | ✓ | ✓ | ✓ |  |  |  |  |
| Hightow-Weidman LB [28] | ✓ | ✓ |  | ✓ |  | ✓ |  |
| Graffigna G [47] |  | ✓ |  | ✓ | ✓ |  |  |
| Levine D [69] |  |  |  | ✓ |  |  |  |
| Ronen K [70] |  |  |  | ✓ |  |  |  |
| Livingood WC [63] |  |  |  | ✓ |  |  |  |
| Van Bruinessen I [66] |  |  |  | ✓ | ✓ | ✓ |  |
| Pernencar C [56] |  |  |  | ✓ |  |  |  |
| Short CE [20] | ✓ |  | ✓ |  |  |  |  |
| Lawrence K [43] |  | ✓ |  | ✓ |  | ✓ |  |
| Wagner B [31] | ✓ |  | ✓ | ✓ |  |  |  |
| Skinner H [42] |  |  |  | ✓ |  |  |  |
| van Hierden Y [57] | ✓ |  |  | ✓ |  |  |  |
| Schroeer C [44] |  | ✓ |  | ✓ |  | ✓ |  |
| Rai T [38] | ✓ |  |  | ✓ | ✓ | ✓ |  |
| Nurmi J [26] | ✓ |  |  | ✓ | ✓ | ✓ |  |
| Mustafa AS [46] |  | ✓ |  | ✓ |  | ✓ |  |
| Morisson J [49] |  |  | ✓ | ✓ |  | ✓ |  |
| Partridge SR [61] |  |  |  | ✓ |  | ✓ |  |
| Mauka W [55] |  |  |  | ✓ |  |  |  |
| Solomon M [48] | ✓ | ✓ | ✓ | ✓ |  | ✓ |  |
| Nitsch M [27] | ✓ |  |  | ✓ |  | ✓ |  |
| Sucala M [65] |  |  |  | ✓ |  | ✓ |  |
| Laidlaw R [58] |  |  |  | ✓ |  |  |  |
| Toefy Y [62] |  |  |  | ✓ |  | ✓ |  |
| Njie-Carr VPS [64] |  |  |  | ✓ |  | ✓ |  |
| Myneni S [18] | ✓ | ✓ | ✓ | ✓ | ✓ |  |  |
| Musso M [37] | ✓ |  |  | ✓ |  |  |  |
| Milward J [10] | ✓ | ✓ |  | ✓ |  | ✓ |  |
| Saleem M [22] | ✓ |  | ✓ | ✓ |  | ✓ |  |
| Hawkes RE [32] | ✓ |  | ✓ |  | ✓ | ✓ |  |
| Kelders SM [17] | ✓ | ✓ | ✓ |  |  |  |  |
| Turcotte S [73] |  |  |  |  | ✓ | ✓ |  |
| Grieve N [29] | ✓ |  |  |  |  | ✓ |  |
| Milne-Ives M [35] | ✓ |  | ✓ | ✓ |  |  |  |
| Mair JL [52] |  |  |  | ✓ | ✓ | ✓ |  |
| White BK [36] | ✓ | ✓ | ✓ |  |  |  |  |
| Giovanelli A [41] | ✓ |  | ✓ | ✓ |  |  |  |
| Isakadze N [53] |  |  |  |  | ✓ | ✓ |  |
| Dederichs M [59] |  |  |  | ✓ | ✓ | ✓ |  |
| Ho TQA [51] | ✓ |  | ✓ | ✓ | ✓ | ✓ |  |
