# Supplementary material for: Engagement of Users in Digital Health Applications: Scoping Review
Source: JMIR Mhealth Uhealth. 2026 May 15;14:e66002. doi: 10.2196/66002 (PMC13179053; doi:10.2196/66002)

**Conceptual Synthesis of the Determinants Influencing User Engagement in Digital Health Interventions, as Identified in This Review**

This figure proposed conceptual framework synthesizes main determinants of user engagement in digital health interventions, as identified in this present review.


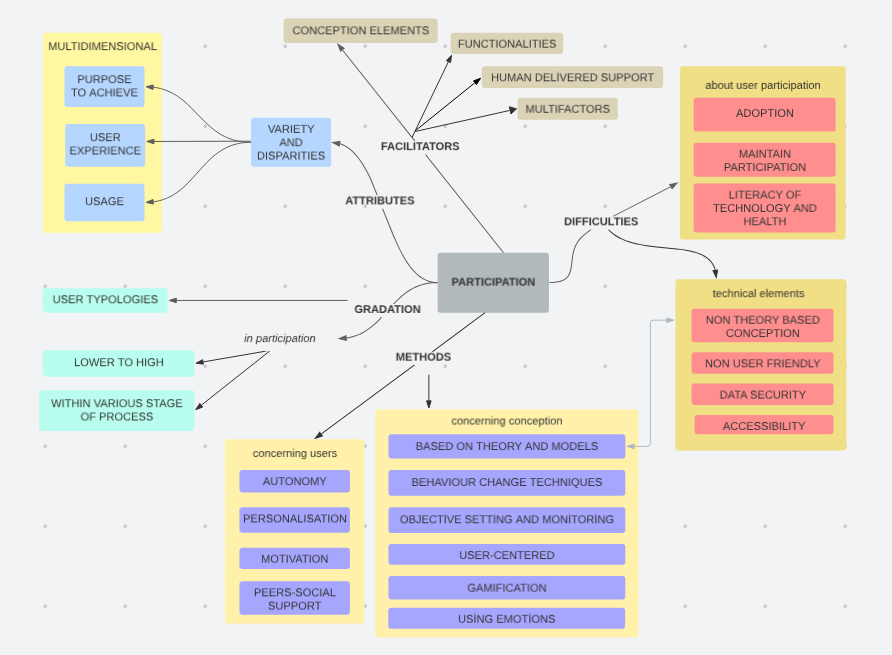

Supplement: Multimedia Appendix 4 [file mhealth-v14-e66002-s004.docx]
